# Supplementary material for: The causal role of circulating inflammatory markers in osteoporosis: a bidirectional Mendelian randomized study
Source: Front Immunol. 2024 Jul 18;15:1412298. doi: 10.3389/fimmu.2024.1412298 (PMC11291241; doi:10.3389/fimmu.2024.1412298)
Supplement: Supplementary file 1 [file DataSheet_1.zip › supplementary material/OSTEOPOROSIS-CIM/GCST90274846.tsv.gz_forest.pdf]

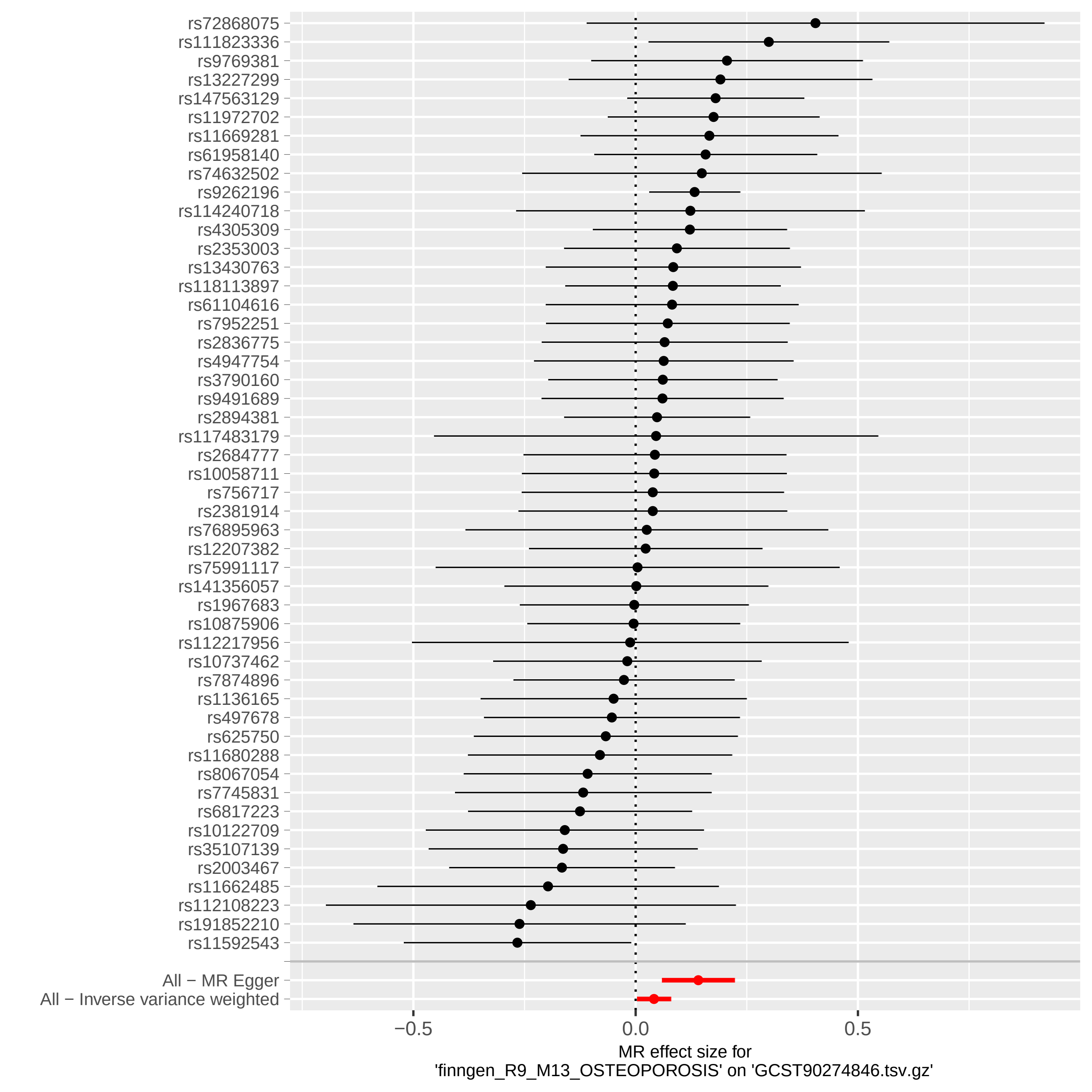
rs72868075
rs111823336
rs9769381
rs13227299
rs147563129
rs11972702
rs11669281
rs61958140
rs74632502
rs9262196
rs114240718
rs4305309
rs2353003
rs13430763
rs118113897
rs61104616
rs7952251
rs2836775
rs4947754
rs3790160
rs9491689
rs2894381
rs117483179
rs2684777
rs10058711
rs756717
rs2381914
rs76895963
rs12207382
rs75991117
rs141356057
rs1967683
rs10875906
rs112217956
rs10737462
rs7874896
rs1136165
rs497678
rs625750
rs11680288
rs8067054
rs7745831
rs6817223
rs10122709
rs35107139
rs2003467
rs11662485
rs112108223
rs191852210
rs11592543
All − MR Egger
All − Inverse variance weighted
−0.5
0.0
0.5
MR effect size for
'finngen_R9_M13_OSTEOPOROSIS' on 'GCST90274846.tsv.gz'
